# Aging Promotes Mitochondria-Mediated Apoptosis in Rat Hearts

**DOI:** 10.3390/life10090178

**Published:** 2020-09-05

**Authors:** Mi-Hyun No, Youngju Choi, Jinkyung Cho, Jun-Won Heo, Eun-Jeong Cho, Dong-Ho Park, Ju-Hee Kang, Chang-Ju Kim, Dae Yun Seo, Jin Han, Hyo-Bum Kwak

**Affiliations:** 1 Department of Biomedical Science, Program in Biomedical Science & Engineering, Department of Kinesiology, Inha University, Incheon 22212, Korea; 77nodaji@hanmail.net (M.-H.N.); gjwnsdnjs03@naver.com (J.-W.H.); cejeong97@naver.com (E.-J.C.); dparkosu@inha.ac.kr (D.-H.P.); 2 Institute of Sports and Arts Convergence, Inha University, Incheon 22212, Korea; choiyoungju0323@gmail.com (Y.C.); lovebuffalo@gmail.com (J.C.); johykang@inha.ac.kr (J.-H.K.); 3 Department of Pharmacology, College of Medicine, Inha University, Incheon 22212, Korea; 4 Department of Physiology, College of Medicine, Kyung Hee University, Seoul 02447, Korea; changju@khu.ac.kr; 5 National Research Laboratory for Mitochondrial Signaling, Department of Physiology, College of Medicine, Cardiovascular and Metabolic Disease Center, Inje University, Busan 47392, Korea; sdy925@gmail.com (D.Y.S.); phyhanj@gmail.com (J.H.)

**Keywords:** aging heart, Bcl-2 family, mitochondria, programmed cell death

## Abstract

Aging represents a major risk for developing cardiac disease, including heart failure. The gradual deterioration of cell quality control with aging leads to cell death, a phenomenon associated with mitochondrial dysfunction in the heart. Apoptosis is an important quality control process and a necessary phenomenon for maintaining homeostasis and normal function of the heart. However, the mechanism of mitochondria-mediated apoptosis in aged hearts remains poorly understood. Here, we used male Fischer 344 rats of various ages, representing very young (1 month), young (4 months), middle-aged (12 months), and old (20 months) rats, to determine whether mitochondria-mediated apoptotic signals and apoptosis in the left ventricle of the heart are altered notably with aging. As the rats aged, the extramyocyte space and myocyte cross-sectional area in their left ventricle muscle increased, while the number of myocytes decreased. Additionally, mitochondrion-mediated apoptotic signals and apoptosis increased remarkably during aging. Therefore, our results demonstrate that aging promotes remarkable morphological changes and increases the degree of mitochondrion-mediated apoptosis in the left ventricle of rat hearts.

## 1. Introduction

Aging is commonly characterized by a gradual deterioration of tissue and organ function, and has been identified as the main cause of cardiac dysfunction. Several factors, including mitochondrial dysfunction, genomic instability, defective proteostasis, and epigenetic changes, have been considered important contributors to aging [1,2]. In particular, aging causes significant alterations in the structure and function of the heart, especially in the left ventricle, in addition to increasing cardiac oxidative stress and inflammation [1,3]. Impaired cardiac structure and function with aging result in enhanced susceptibility to cardiovascular diseases (such as heart failure), which can contribute to morbidity and mortality.

Mitochondria are the energy power plants of cells, controlling their fate. They are an important player in various processes and have the ability to maintain metabolic homeostasis and manage aging-related mechanisms [2]. Several studies suggested that mitochondria have evolved to regulate other cellular functions, including those contributing to biological aging, such as the generation of reactive oxygen species (ROS), inflammation, senescence, and resultant necrotic and apoptotic cell death [4]. Our studies have revealed that aging mainly results in damage to the electron transport chain during mitochondrial respiration, due to a decrease in electron transport by complex II-IV, which can promote electron leakage and ROS production [5].

Apoptosis, which is known as programmed cell death, is an essential process for cellular and tissue homeostasis, maintaining cell growth and differentiation, and controlled tissue repair [4,6]. However, excessive apoptosis in the heart induces cardiac dysfunction [7]. In particular, it was reported that reduced heart muscle fiber mass due to apoptosis directly results in the development of myocardial contraction abnormalities, heart failure, and other cardiac conditions [8,9]. The mitochondrion-driven apoptotic pathway, regulated by members of the Bcl-2 family, plays a pivotal regulatory role in aging [10]. An imbalance between the pro-apoptotic proteins Bax and Bid and the anti-apoptotic proteins Bcl-2 and Bcl-xl provokes the opening of the mitochondrial permeability transition pore (mPTP), which, in turn, triggers the translocation of cytochrome c from the mitochondrial intermembrane to the cytoplasm [11]. The released cytochrome c binds to adenosine triphosphate, apoptotic protease-activating factor 1, and pro-caspase 9, which subsequently activates caspase-3, thereby causing DNA fragmentation [12,13,14]. Data from our group and other studies have shown that aging alters the mitochondrial apoptotic pathway in the skeletal and cardiac muscles of rats, resulting in increased Bax/Bcl-2 ratio, cleaved caspase-3 levels, and DNA fragmentation [14,15,16]. However, Nitahara et al. [17] reported that aging has no effect on mitochondria-dependent apoptosis, in particular, the expression of Bax and Bcl-2, in the rat heart. Nevertheless, aging induced cardiomyocyte apoptosis. Despite these findings, the effects of aging on the regulation of mitochondria-mediated apoptosis in the rat heart remain unclear.

In the present study, we aimed to determine whether mitochondria-mediated apoptosis on cardiac muscle would induce greater changes by aging. To distinguish the alterations that occur during biological aging (i.e., growth, development, and aging phases), we investigated the changes in the myocardial structure and mitochondria-mediated apoptotic signaling in the cardiac muscles of male Fischer 344 rats during the four stages of life, based on the lifespan characteristics of this rat strain, i.e., very young (VYG; 1 month old), young (YG; 4 months old), middle-aged (MG; 10 months old), and old (OG; 20 months old). We hypothesized that aging would induce more drastic changes in myocardial structure and apoptosis mediated through the mitochondrial pathway—including alterations in the levels of Bcl-2 family proteins, mPTP opening, cleaved caspase-3, and DNA fragmentation—that correspond to the growth and development phases of the rat heart.

## 2. Materials and Methods

### 2.1. Animal Experiments and Ethical Approval

Forty male Fischer 344 rats were randomly divided into four groups according to age: very young (VYG, 1 month old; n = 10), young (YG, 4 months old; n = 10), middle-aged (MG, 10 months old; n = 10), and old (OG, 20 months old; n = 10). All rats were housed (two per cage) under standardized conditions (20 ± 2 °C; 12 h light:dark cycle). Standard laboratory chow (LabDiet 5L79, Orient Bio, Gyeonggi-Do, Korea) and water were provided ad libitum. The experimental protocol was approved by the Institutional Animal Care and Use Committee of the Kyung Hee University (approval number: KHUASP [SE]-17-089), and was performed in accordance with the animal care guidelines of the ethics committee.

### 2.2. Tissue Preparation

Frozen left ventricle (LV) tissues were homogenized in CETi lysis buffer (pH 7.6; TransLab, Korea) containing a non-ionic detergent, protease inhibitors (4 mM AEBSF, 1 µg/mL benzamidine, 1 µg/mL leupeptin, 1 µg/mL pepstatin, 1 mM EDTA, and 1 mM EGTA), and phosphatase inhibitors (1 mM sodium fluoride, 1 mM sodium orthovanadate, 1 mM beta-glycerophosphate, and 2.5 mM sodium pyrophosphate). The LV tissues were prepared with the OMNI TH115 Tissue Homogenizer (OMNI International, Kennesaw, GA, USA). The samples were centrifuged at 10,000× *g* twice (20 and 10 min, respectively) at 4 °C, and the supernatants were collected for protein analysis.

### 2.3. Hematoxylin and Eosin Staining

LV tissues were first subjected to paraffin-embedding; then, the paraffin-embedded tissue blocks were cut into 5-μm-thick cross-sections, and the sections were placed on glass slides. Next, the sections were deparaffinized by xylene treatment and hydrated in running distilled water for at least 2 min. The samples were stained with hematoxylin for 5 min, rinsed with running tap water, and stained with eosin solution for 2 min. The slides were then rinsed with absolute alcohol. They were then mounted using synthetic resin and dried overnight at room temperature. Images of the section were captured on a microscope of Axioplan 2 (Carl Zeiss, Jena, Germany) and the images were quantified using the ImageJ analysis program (NIH, Bethesda, MD, USA). Two sections of LV in 4 rats per group were analyzed for percentage of extramyocyte space, number of myocytes, and cross-sectional area (CSA). Each section was targeted to the endocardial region. The average myocyte count of the sections of the group was calculated per 100,000 μm^2^ area, and the mean myocyte CSA for the left ventricular histological section is square micrometers.

### 2.4. Western Immunoblotting

The levels of proteins involved in apoptotic signaling—Bax, Bcl-2, and cleaved caspase-3—were determined via Western blotting. Total protein (20 µg) from homogenized LV tissues was denatured at 95 °C for 5 min. The samples were loaded onto 10–12% SDS–polyacrylamide gels and resolved by electrophoresis at 110 V for 2 h. The proteins were electro-transferred onto a nitrocellulose membrane (Pall Corporation, Port Washington, NY, USA) for 1 h at 260 mA. Thereafter, the membranes were stained with Ponceau S (Sigma-Aldrich, St. Louis, MO, USA) to ensure equal loading and protein transfer of all samples. The membranes were blocked with 5% skim milk in TBS buffer with 0.1% Tween-20 (TBST) for 2 h at room temperature, washed thrice for 10 min with TBST, and incubated at 4 °C for 12 h, with the following primary antibodies (diluted in TBS): anti-Bax (1:1000; Santa Cruz Biotechnology, Dallas, TX, USA), anti-Bcl-2 (1:1000; Santa Cruz Biotechnology), and anti-cleaved caspase-3 antibodies (1:500; Cell Signaling Technology, Beverly, MA, USA). The membranes were then washed thrice for 10 min with TBST. Thereafter, the membranes were slowly incubated on a shaker for 1 h with horseradish peroxidase-conjugated anti-mouse or anti-rabbit secondary antibodies (1:3000 in TBST; Santa Cruz) at room temperature. After washing with TBST, protein bands were developed using an enhanced chemiluminescence detection kit (Thermo Scientific, Waltham, MA, USA). Relative protein quantification was performed by densitometry analysis using the Image-Pro Plus software (Media Cybernetics, Rockville, MD, USA).

### 2.5. Mitochondrial Permeability Transition Pore (mPTP) Opening Sensitivity

The mitochondrial Ca^2+^-retention capacity is commonly used to evaluate the susceptibility of mPTP opening sensitivity, as previously reported [5]. The mPTP opening was determined through the Ca^2+^-retention capacity graph. The value of the Ca^2+^-retention capacity of the control group (VYG) was calculated indirectly by dividing the other groups, and comparisons were expressed as percentage values.

### 2.6. Immunohistochemistry

LV tissues were first embedded in paraffin; these paraffin blocks were cut into 5-μm-thick sections, which were placed onto slides and washed thrice for 3 min with phosphate buffered saline (PBS) solution and incubated in 3% hydrogen peroxide for 30 min. Afterwards, the slides were washed thrice with PBS and incubated in PBS with 10% goat serum and 1% bovine serum albumin (BSA) at room temperature for 2 h. The sections were incubated overnight at 4 °C with anti-cleaved caspase-3 antibodies (1:600; Cell Signaling Technology) diluted in PBS with 1% BSA. The slides were gently washed thrice with PBS and incubated in anti-rabbit secondary antibodies (1:1000; Santa Cruz Biotechnology) at room temperature for 1 h, and washed thrice with PBS. The ABC reagent kit (1:100; Vector Laboratories, Burlingame, CA, USA) was used to amplify the protein signals, and the sections were visualized by staining with 0.03% diaminobenzidine (a chromogenic substrate) at room temperature. The slides were stained and dehydrated via a hematoxylin control method, and then cover-slipped. The cleaved caspase-3–positive cells were quantified in the LV tissue sections using the ImageJ software (version 1.52a; NIH, Bethesda, MD, USA).

### 2.7. Terminal Deoxynucleotidyl Transferase-Mediated dUTP Nick-End Labeling (TUNEL Assay)

To identify DNA fragmentation, we performed TUNEL staining using an ApopTag Plus Peroxidase In Situ Apoptosis Detection Kit (Intergen Company, Purchase, NY, USA), according to the manufacturer’s instructions. The slides were soaked in an ethanol-acetic acid solution (2:1 v/v dilution), placed in a freezer for 5 min, and washed thrice with PBS. Sections were sequentially incubated with 0.5% Triton X-100, Protease K (100 mg/mL), 3% hydrogen peroxide, and TUNEL reaction mixture, which was used to rinse the samples after each step. The staining was performed using horseradish peroxidase-tagged antibodies and 0.03% diaminobenzidine, with counterstaining using the Nissl dye. TUNEL-positive myonuclei were counted in two sections of LV in 4 rats per group, and the averages were calculated as percentages of the total number of labeled myonuclei.

### 2.8. Statistical Analysis

Data were expressed as the means ± standard errors of means (SEMs). The differences among the four study groups were analyzed by one-way analysis of variance (ANOVA) with post-hoc Tukey’s test. Statistical significance was considered at *p* < 0.05.

## 3. Results

### 3.1. Effects of Aging on Cardiac Muscle Morphology

**Figure 1:**
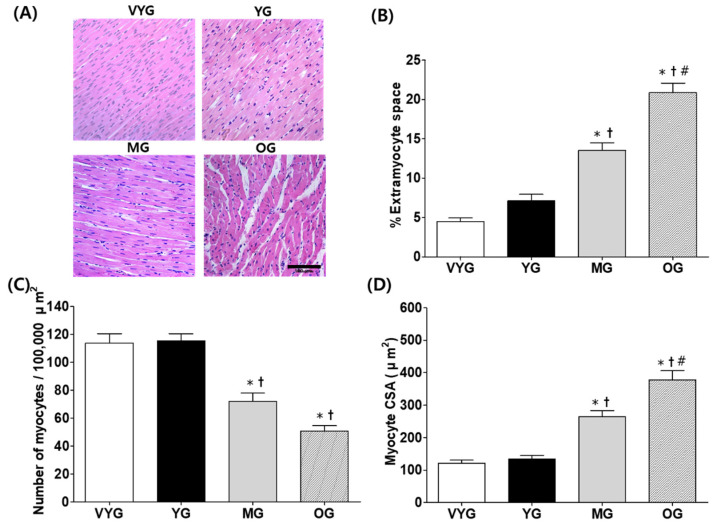
(**A**) Representative histological cross-sections of the left ventricle (LV) tissues of rats from the very young (VYG), young (YG), middle-aged (MG), and old (OG) groups stained with hematoxylin and eosin (amplification: 40×). Unstained areas indicate extramyocyte space. (**B**) Quantification of the percentage of extramyocyte space. (**C**) Number of myocytes per 100,000 µm^2^ in the LV tissues. (**D**) Myocyte cross-sectional area (CSA) of LV tissue histological sections, in square micrometers. The scale bar indicates 100 μm. Data presented as the means ± SEMs. * *p* < 0.05 vs. VYG. † *p* < 0.05 vs. YG. # *p* < 0.05 vs. MG.

LV cross-sections were stained with hematoxylin and eosin to assess their morphology. Cardiac muscle remodeling was determined by measuring extramyocyte space, extent of apparent fibrosis, myocyte cross-sectional area (CSA), and the number of myocytes per 100,000 µm^2^ (Figure 1A). We found that the samples from the OG group showed more extensive cardiac muscle remodeling compared with those from the other groups. No difference in the percentage of extramyocyte space in the samples from the VYG and YG groups was observed, while the samples from the OG group showed significantly more extramyocyte space than those from the VYG, YG, and MG groups; furthermore, the samples from the MG group had more extramyocyte space than those from the VYG and YG groups (mean ± SEM; VYG: 4.48 ± 0.47%; YG: 7.13 ± 0.83%; MG: 13.51 ± 0.97%; OG: 20.85 ± 1.23%)) (*p* < 0.05 in all comparisons, Figure 1B). The number of myocytes per 100,000 µm^2^ in the LV tissues was significantly lower in case of the samples from the MG and OG groups compared with those from the VYG and YG groups (mean ± SEM; VYG: 113.70 ± 6.79; YG: 115.38 ± 5.10; MG: 72.17 ± 5.83; OG: 50.86 ± 3.83)) (*p* < 0.05 in all comparisons, Figure 1C). In contrast, the mean myocyte CSA in the combined myocardial and endocardial regions was significantly higher in the OG group than in the VYG, YG, and MG groups (mean ± SEM; 377.66 ± 28.38 µm^2^ vs. 120.94 ± 9.34, 134.04 ± 10.79, and 264.22 ± 18.90 µm^2^, respectively) (*p* < 0.05 in all comparisons, Figure 1D), and higher in the MG rats than in the VYG and YG animals (mean ± SEM; 264.22 ± 18.90 µm^2^ vs. 120.94 ± 9.34 and 134.04 ± 10.79 µm^2^, respectively) (*p* < 0.05 in all comparisons, Figure 1D).

### 3.2. Effects of Aging on Mitochondria-Mediated Apoptotic Signaling in Cardiac Muscles

**Figure 2:**
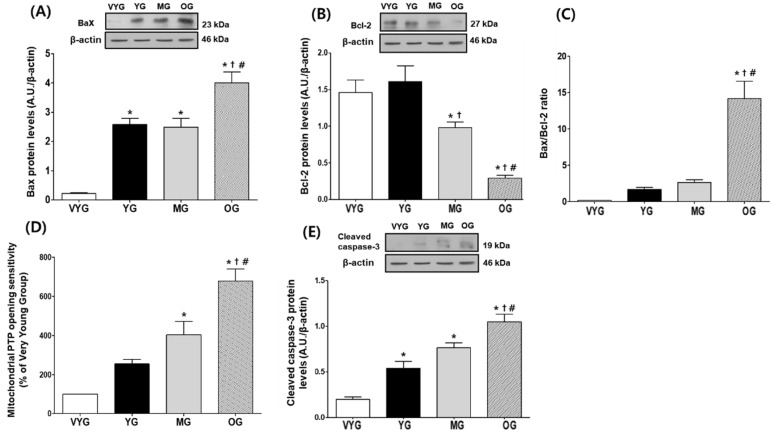
Mitochondria-mediated apoptotic signaling in the left ventricle (LV) tissues of rats from the very young (VYG), young (YG), middle-aged (MG), and old (OG) groups. (**A**) Bax levels detected by Western blotting with actin as the normalization control. (**B**) Representative Western blot results and relative quantification of Bcl-2 levels in LV tissues. (**C**) Representative Bax/Bcl-2 ratios in LV tissues. (**D**) Representative mPTP opening sensitivity with that of the VYG samples as the normalization control. (**E**) Cleaved caspase-3 levels detected by Western blotting, with actin as the normalization control. Data are presented as the means ± SEMs. * *p* < 0.05 vs. VYG. † *p* < 0.05 vs. YG. # *p* < 0.05 vs. MG.

The expression of the pro-apoptotic Bax protein increased by 1960%, 78%, and 69% in the samples from the OG group (4.12 ± 0.35), compared with that in samples from the VYG (0.20 ± 0.04), YG (2.32 ± 0.34), and MG groups (2.44 ± 0.31), respectively (*p* < 0.05 in all comparisons, Figure 2A). The samples from the YG and MG groups showed similar Bax levels (*p* > 0.05). In contrast, the levels of the anti-apoptotic Bcl-2 protein were reduced by 80%, 82%, and 70% in the samples from the OG group (0.29 ± 0.04), compared with those in the samples from the VYG (1.46 ± 0.17), YG (1.61 ± 0.22), and MG (0.98 ± 0.08) groups, respectively (*p* < 0.05 in all comparisons, Figure 2B), while the samples from the VYG and YG groups showed similar Bcl-2 levels (*p* > 0.05). The Bax/Bcl-2 ratio, which is important during the early stage of mitochondria-mediated apoptosis, increased more significantly in the samples from the OG group than in those from the VYG, YG, and MG groups (*p* < 0.05, Figure 2C). Analysis of mPTP opening sensitivity in the VYS group was set to 100.00; the mPTP opening sensitivity was increased by 154%, 303%, and 587% in the samples from the OG group (687.19 ± 60.83), compared with that in the samples from the VYG (100.00), YG (254.16 ± 23.76), and MG groups (403.55 ± 68.45), respectively (*p* < 0.05 in all comparisons, Figure 2D). Furthermore, the mPTP opening sensitivity was higher in the samples from the MG group than in those from the VYG group (*p* < 0.05, Figure 2D), while no significant difference was seen between the mPTP opening sensitivity in the samples from the VYG and YG groups (100.00 vs. 254.16 ± 23.76; Figure 2D). Analysis of the expression of cleaved caspase-3, which is present downstream to the Bcl-2 family in the mitochondria-mediated apoptotic pathway, revealed that its levels were significantly higher in OG rats (1.05 ± 0.08) than in the VYG (0.20 ± 0.03), YG (0.54 ± 0.08), and MG (0.76 ± 0.05) rats (*p* < 0.05 in all comparisons, Figure 2E). Furthermore, the levels of cleaved caspase-3 were higher in the samples from the YG and MG groups than in those from the VYG group (*p* < 0.05 in both comparisons, Figure 2E).

### 3.3. Effects of Aging on Cleaved Caspase-3-Positive Cells and TUNEL-Positive Myonuclei in Cardiac Muscles

**Figure 3:**
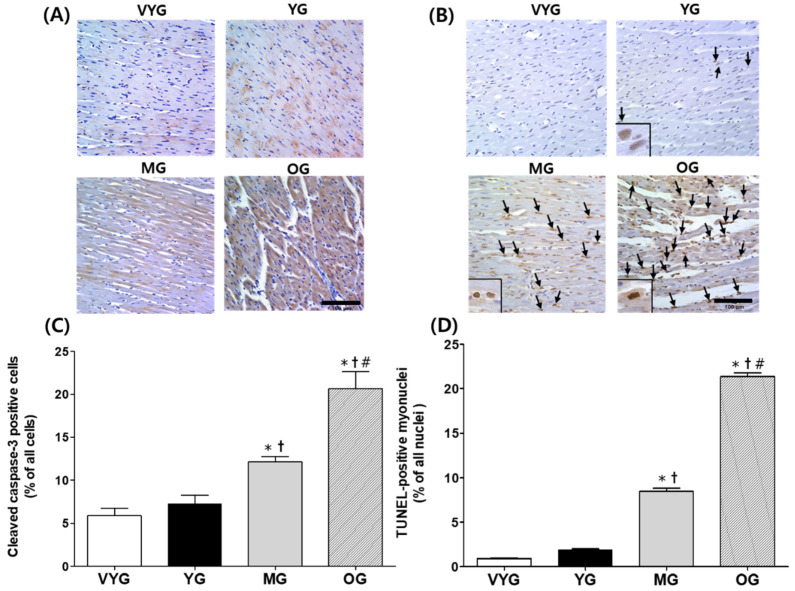
Cleaved caspase-3–positive cells and TUNEL-positive myonuclei in the left ventricle (LV) tissues of rats from the very young (VYG), young (YG), middle-aged (MG), and old (OG) groups (scale bar: 100 μm and magnification: 40×). (**A**) Representative photographs of LV tissue sections stained with anti-cleaved caspase-3 antibodies (**B**) TUNEL staining images in which brown-stained regions represent TUNEL-positive myonuclei. Apoptotic myonuclei are indicated by arrows which are typically enlarged in the lower left corner. (**C**) Quantification of cleaved caspase-3–positive cells via immunohistochemical staining. (**D**) Quantification of TUNEL-positive myonuclei. Data are presented as the means ± SEMs. * *p* < 0.05 vs. VYG. † *p* < 0.05 vs. YG. # *p* < 0.05 vs. MG.

The number of cleaved caspase-3-positive cells increased by 250% and 185% in the samples from the OG group (20.64 ± 1.96), compared with that in samples from the VYG (5.90 ± 0.85) and YG groups (7.24 ± 1.00), respectively (*p* < 0.05 in both comparisons; Figure 3A,C). Additionally, the number of TUNEL-positive myonuclei in the cardiac muscles of OG rats was significantly higher than that in the cardiac muscles of the VYG (by 2196%), YG (by 810%), and MG (by 107%) rats (mean ± SEM; 21.35 ± 0.42 vs. 0.93 ± 0.05, 1.92 ± 0.08, and 8.46 ± 0.35, respectively) (*p* < 0.05 in all comparisons; Figure 3B,D). The number of TUNEL-positive myonuclei was higher in the samples from the MG group than in those from the VYG and YG groups (*p* < 0.05 in both comparisons, Figure 3B,D). However, the samples from the VYG and YG groups did not show significant differences in the numbers of cleaved caspase-3-positive cells and TUNEL-positive myonuclei (*p* > 0.05).

## 4. Discussion

The main findings of this study were as follows: (i) cardiac muscle remodeling, assessed based on morphological changes in tissues, increased with advancing age; (ii) mitochondria-dependent apoptotic signaling (including Bax/Bcl-2 ratio, mPTP opening sensitivity, and cleaved caspase-3 protein levels) remarkably increased with advancing age; and (iii) apoptosis (including numbers of cleaved caspase-3-positive cells and TUNEL-positive myonuclei) also increased with advancing age. These results reveal that aging induces significant alterations in the myocardial structure and mitochondria-mediated apoptotic signaling in the rat heart, and that these changes are more drastic during the old-age phase and not in the developing-age phase.

To our knowledge, this is the first report regarding the changes in the myocardial structure and mitochondria-mediated apoptotic signaling, which occurs via Bcl-2 family proteins, in rat cardiac muscles throughout their lifespan—including each phase of growth (very young vs. young), development (young vs. middle-aged), and aging (middle-aged vs. old). As age-related changes in mitochondria-mediated apoptotic signaling are debatable, determination of such signals, as well as the myocardial structure at different ages will likely provide valuable information on the cellular and molecular mechanisms underlying aging in the heart.

Several studies using models of young and old animals have shown that cardiac remodeling and function deteriorate with age [18,19,20]. Consistent with previous findings, we also observed significant cardiac morphological changes, such as increased extramyocyte space and myocyte CSA, in aged rats, compared with younger animals during the growth and development phases. However, these changes seemed gradual between the development and aging phases. Aging is generally perceived as the most drastic morphological change in the cardiac muscle, with more subtle and progressive changes in the growth and development phases. Progressive aging of the heart is caused by the excessive deposition of extracellular matrix (ECM) elements, such as collagen and fibronectin, triggered by the uncontrolled activation of the fibrosis pathway and suppression of anti-fibrosis signals, which can lead to cardiac fibrosis [21]. Hence, aging may cause an excessive accumulation of ECM components in cardiomyocytes, leading to heart failure caused by pathological mechanisms, including diastolic decline and cardiac hypertrophy [22,23]. Our results suggest that aging is the main cause of increased extramyocyte space and myocyte CSA, and the reduced number of cardiomyocytes.

Mitochondrial dysfunction leads to an imbalance of Bax and Bcl-2 levels, which will, in turn, activate caspase-3, a pivotal protein involved in mitochondria-mediated apoptosis [14,24]. Oxidative stress is also known to cause the release of Bax into the cytoplasm, which will promote the mPTP opening sensitivity and the activation of caspase-9 and caspase-3. These signals eventually result in DNA fragmentation and programmed cell death [12,16,25]. We recently found increased mitochondrial hydrogen peroxide production with aging in rat cardiac muscles [5]. Interestingly, the current study revealed that the Bax/Bcl-2 ratio was markedly higher in the cardiac muscles of older rats, indicating that the aging phase was related to early-stage mitochondria-mediated apoptosis. Moreover, progressively increased mPTP opening sensitivity and the cleavage of caspase-3 were seen during the aging process, and they occurred more prominently in the aging phase. We believe that apoptosis is primarily driven by mitochondrial ROS accumulation and mPTP opening. Therefore, it is possible that aging could enhance mitochondria-mediated apoptotic signaling [26,27], which will contribute further towards cardiac muscle apoptosis.

Additionally, we found that the number of TUNEL-positive myonuclei, which is an apoptosis marker, was affected by aging, especially during the development and aging phases. Fannin et al. [28] investigated the mitochondria-mediated apoptotic pathway in aging female F344xBN rats and suggested that aging was associated with increases in the Bax/Bcl-2 ratio, caspase-3 activation, and the number of TUNEL-positive myonuclei. Apoptosis has an important homeostatic role in normal, healthy hearts; however, excessive apoptosis leads to pathological, life-threatening heart dysfunction during the aging process [29]. We did not measure the cardiac function in addition to it being relative to aging; however, apoptosis might have affected cardiac function [30]. Further studies are necessary to better understand the role of mitochondria-mediated apoptosis in aging-induced cardiac dysfunction.

The present study has some limitations. First, we did not include a histochemistry analysis of mitochondrial respiratory chain complexes and mitochondrial ultrastructure examination in heart tissues that can provide important information to prove the relationship between mitochondrial dysfunction and aging. Second, since female hormones (e.g., estrogen) regulate mitochondrial function [31], we used only male rats, including very young (1 months), young (4 months), middle-aged (10 months), and old (20 months) rats as animal models in this study.

## 5. Conclusions

This study showed that aging induces cardiac muscle remodeling in rats, promoting increases in extramyocyte space and the CSA and reducing the number of myocytes, which are key features involved in cardiac fibrosis. Additionally, aging induces mitochondria-mediated apoptotic signals (Bax expression, increased Bax/Bcl-2 ratio and mPTP opening sensitivity, and the activation of caspase-3) and apoptosis (TUNEL-positive myonuclei) in cardiac muscles. These results provide strong evidence supporting our hypothesis that aging would cause drastic changes in the myocardial structure, mitochondria-mediated apoptotic signaling, and DNA fragmentation in rat hearts. Nevertheless, morphological and molecular changes in cardiac muscles are substantially less prominent during the growth and development phases of the lifespan of rats.
